# Supplementary material for: Dirt floors and domestic animals are associated with soilborne exposure to antimicrobial resistant E. coli in rural Bangladeshi households
Source: bioRxiv. 2025 Feb 23:2025.02.21.639507. Preprint. [Version 1] doi: 10.1101/2025.02.21.639507 (PMC11870552; doi:10.1101/2025.02.21.639507)
Supplement: Supplement 1 [file media-1.pdf]

Supplemental Information

Dirt floors and domestic animals are associated with soilborne exposure to antimicrobial resistant *E. coli* in rural Bangladeshi households

Table S1. Animal ownership and management practices 1

Table S2. Animal ownership vs. roaming 2

Table S3. Prevalence of generic and cefotaxime-resistant *E. coli* on floor swabs by animal ownership and management practices 3

Table S4. Log10-transformed most probable number (MPN) of generic and cefotaxime-resistant *E. coli* and relative abundance of cefotaxime-resistant *E. coli* on floor swabs by animal ownership and management practices 4

Table S5. Number of animals owned by quartiles of generic and cefotaxime-resistant *E. coli* counts and percent abundance of cefotaxime-resistant *E. coli* on floor swabs 5

Table S6. Prevalence and log10-transformed most probable number (MPN) of *E. coli* on child hands by animal ownership and management practices 6

Table S7. Number of animals owned by quartiles of *E. coli* counts on child hands 7

Table S8. *E. coli* prevalence and abundance on floor swabs by frequency of animal roaming 8

Table S9. *E. coli* prevalence and abundance on child hands by frequency of animal roaming 9

Table S10. *E. coli* prevalence and abundance on floor swabs by animal cohabitation intensity 10

Table S11. *E. coli* prevalence and abundance on child hands by animal cohabitation intensity 11

Table S12. *E. coli* prevalence and abundance on floor swabs and child hands by cross-categories of household floor type and animal ownership 12

Table S1. Animal ownership and management practices

	All households (N=49)	Households with soil floors (N=28)	Households with concrete floors (N=21)
**Household owns at least 1, % (n)**			
Chicken/duck	59.2 (29)	71.4 (20)	42.9 (9)
Cattle/buffalo	49.0 (24)	57.1 (16)	38.1 (8)
Goat/sheep	42.9 (21)	53.6 (15)	28.6 (6)
Any animal	71.4 (35)	78.6 (22)	61.9 (13)
**Compound owns at least 1, % (n)**			
Chicken/duck	63.3 (31)	75.0 (21)	47.6 (10)
Cattle/buffalo	59.2 (29)	64.3 (18)	52.4 (11)
Goat/sheep	46.9 (23)	57.1 (16)	33.3 (7)
Any animal	77.6 (38)	82.1 (23)	71.4 (15)
**Number of animals in household, mean (SD)**			
Chicken/duck	12.8 (24.0)	11.5 (12.6)	14.5 (34.2)
Cattle/buffalo	1.7 (2.4)	2.3 (2.8)	0.9 (1.5)
Goat/sheep	1.7 (3.1)	1.9 (2.3)	1.5 (3.9)
Any animal	16.2 (26.7)	15.7 (15.1)	16.9 (37.4)
**Number of animals in compound, mean (SD)**			
Chicken/duck	14.1 (24.1)	13.9 (12.9)	14.4 (34.2)
Cattle/buffalo	2.2 (2.9)	2.8 (3.3)	1.5 (2.1)
Goat/sheep	1.9 (3.1)	2.0 (2.2)	1.8 (4.0)
Any animal	18.2 (26.5)	18.7 (15.4)	17.6 (36.9)
**Animals ever roam free in household:**			
Chicken/duck	61.2 (30)	75.0 (21)	42.9 (9)
Cattle/buffalo	26.5 (13)	35.7 (10)	14.3 (21)
Goat/sheep	34.7 (17)	50.0 (14)	14.3 (3)
Any animal	63.3 (31)	75.0 (21)	47.6 (10)
**Animals ever roam free in compound:**			
Chicken/duck	65.3 (32)	75.0 (21)	52.4 (11)
Cattle/buffalo	28.6 (14)	32.1 (9)	23.8 (5)
Goat/sheep	42.9 (21)	57.1 (16)	23.8 (5)
Any animal	73.5 (36)	82.1 (23)	61.9 (13)
**Animals kept inside home at night [a]:**			
Chicken/duck	6.9 (2)	10.0 (2)	0.0 (0)
Cattle/buffalo	12.5 (3)	18.8 (3)	0.0 (0)
Goat/sheep	38.1 (8)	53.3 (8)	0.0 (0)
Any animal	25.7 (9)	40.9 (9)	0.0 (0)
**Animal feces observed on floor [a]:**			
Chicken/duck	4.1 (2)	7.1 (2)	0.0 (0)
Cattle/buffalo	4.1 (2)	7.1 (2)	0.0 (0)
Goat/sheep	2.0 (1)	3.6 (1)	0.0 (0)
Any animal	8.2 (4)	14.3 (4)	0.0 (0)

Table S2. Animal ownership vs. roaming

	Yes	Roams in household	Roams in compound	No	Roams in household	Roams in compound
**Household owns:**	**N**	**% (n)**	**% (n)**	**N**	**% (n)**	**% (n)**
Chickens	29	96.6 (28)	100.0 (29)	20	10.0 (2)	15.0 (3)
Cows	24	45.8 (11)	50.0 (12)	25	8.0 (2)	8.0 (2)
Goat	21	76.2 (16)	95.2 (20)	28	3.6 (1)	3.6 (1)
Any animal	35	82.9 (29)	94.3 (33)	14	14.3 (2)	21.4 (3)
**Compound owns:**	**N**	**% (n)**	**% (n)**	**N**	**% (n)**	**% (n)**
Chickens	31	93.6 (29)	100.0 (31)	18	5.6 (1)	5.6 (1)
Cows	29	44.8 (13)	48.3 (14)	20	0.0 (0)	0.0 (0)
Goat	23	73.9 (17)	91.3 (21)	26	0.0 (0)	0.0 (0)
Any animal	38	79.0 (30)	92.1 (35)	11	9.1 (1)	9.1 (1)

Table S3. Prevalence of generic and cefotaxime-resistant *E. coli* on floor swabs by animal ownership and management practices

			**Generic *E. coli***			**Cefotaxime-resistant *E. coli***		
	**N**		**Prevalence % (n)**		**Chi2**	**Prevalence % (n)**		**Chi2**
	**Yes**	**No**	**Yes**	**No**	**p-value**	**Yes**	**No**	**p-value**
**Household owns at least 1:**								
Chicken/duck	29	20	100.0 (29)	85.0 (17)	0.03	79.3 (23)	55.0 (22)	0.07
Cattle/buffalo	24	25	100.0 (24)	88.0 (22)	0.08	79.2 (19)	60.0 (15)	0.15
Goat/sheep	21	28	100.0 (21)	89.3 (25)	0.12	76.2 (16)	64.3 (18)	0.37
Any animal	35	14	100.0 (35)	78.6 (11)	0.005	77.1 (27)	50.0 (14)	0.06
**Compound owns at least 1:**								
Chicken/duck	31	18	100.0 (31)	83.3 (15)	0.02	83.9 (26)	44.4 (8)	0.004
Cattle/buffalo	29	20	100.0 (29)	85.0 (17)	0.03	79.3 (23)	55.0 (11)	0.07
Goat/sheep	23	26	95.7 (22)	92.3 (24)	0.63	73.9 (17)	65.4 (17)	0.52
Any animal	38	11	97.4 (37)	81.8 (9)	0.06	79.0 (30)	36.4 (4)	0.007
**Animals ever roam free in household:**								
Chicken/duck	30	19	100.0 (30)	84.2 (16)	0.03	80.0 (24)	52.6 (10)	0.04
Cattle/buffalo	13	36	100.0 (13)	91.7 (33)	0.28	84.6 (2)	63.9 (23)	0.17
Goat/sheep	17	32	100.0 (17)	90.6 (29)	0.19	82.4 (14)	62.5 (20)	0.15
Any animal	31	18	100.0 (31)	83.3 (15)	0.02	80.7 (25)	50.0 (9)	0.03
**Animals ever roam free in compound:**								
Chicken/duck	32	17	100.0 (32)	82.4 (14)	0.01	81.3 (26)	47.1 (8)	0.01
Cattle/buffalo	14	35	100.0 (14)	91.4 (32)	0.26	71.4 (25)	64.3 (9)	0.62
Goat/sheep	21	28	100.0 (21)	89.3 (25)	0.12	76.2 (16)	64.3 (18)	0.37
Any animal	36	13	100.0 (36)	76.9 (10)	0.003	77.8 (28)	46.2 (6)	0.03
**Animals kept inside home at night [a]:**								
Chicken/duck	2	27	100.0 (2)	100.0 (2)	--	100.0 (2)	77.8 (21)	0.62
Cattle/buffalo	3	21	100.0 (3)	100.0 (21)	--	100.0 (3)	76.2 (16)	0.48
Goat/sheep	8	13	100.0 (8)	100.0 (13)	--	100.0 (8)	61.5 (8)	0.06
Any animal	9	26	100.0 (9)	100.0 (26)	--	100.0 (9)	69.2 (18)	0.07
**Animal feces observed on floor [a]:**								
Chicken/duck	2	47	100.0 (2)	93.6 (44)	0.88	100.0 (2)	68.1 (32)	0.48
Cattle/buffalo	2	47	100.0 (2)	93.6 (44)	0.88	100.0 (2)	68.1 (32)	0.48
Goat/sheep	1	48	100.0 (1)	93.8 (45)	0.94	100.0 (1)	68.8 (33)	0.69
Any animal	4	45	100.0 (4)	93.3 (42)	0.77	100.0 (4)	66.7 (30)	0.22

[a] Fisher's exact test performed because of small cells.

Table S4. Log10-transformed most probable number (MPN) of generic and cefotaxime-resistant *E. coli* and relative abundance of cefotaxime-resistant *E. coli* on floor swabs by animal ownership and management practices

			**Generic *E. coli***			**Cefotaxime-resistant *E. coli***			**% abundance**		
	**N**		**Log10 MPN Mean (SD)**		**MW**	**Log10 MPN Mean (SD)**		**MW**	**Mean (SD)**		**MW**
	**Yes**	**No**	**Yes**	**No**	**p-value**	**Yes**	**No**	**p-value**	**Yes**	**No**	**p-value**
**Household owns at least 1:**											
Chicken/duck	29	20	4.3 (1.3)	3.2 (1.6)	0.02	2.8 (1.3)	2.0 (1.1)	0.03	13.3 (26.5)	5.6 (14.4)	0.26
Cattle/buffalo	24	25	4.2 (1.4)	3.5 (1.5)	0.07	2.8 (1.3)	2.2 (1.2)	0.06	16.9 (30.1)	3.5 (1.2)	0.30
Goat/sheep	21	28	4.2 (1.4)	3.6 (2.5)	0.14	2.8 (1.4)	2.2 (1.1)	0.14	14.9 (30.4)	6.8 (13.5)	0.64
Any animal	35	14	4.2 (1.4)	3.1 (1.6)	0.03	2.8 (1.3)	1.8 (0.8)	0.02	13.5 (25.6)	1.1 (1.6)	0.09
**Compound owns at least 1:**											
Chicken/duck	31	18	4.3 (1.2)	3.1 (1.6)	0.01	2.8 (1.3)	2.0 (1.1)	0.03	12.7 (25.7)	6.0 (15.4)	0.10
Cattle/buffalo	29	20	4.2 (1.3)	3.4 (1.7)	0.11	2.7 (1.3)	2.3 (1.3)	0.19	14.2 (28.0)	4.2 (6.0)	0.54
Goat/sheep	23	26	4.1 (1.5)	3.6 (1.5)	0.25	2.8 (1.4)	2.2 (1.1)	0.15	15.0 (30.0)	6.4 (13.6)	0.43
Any animal	38	11	4.1 (1.4)	3.1 (1.6)	0.06	2.7 (1.3)	1.8 (0.9)	0.03	12.9 (25.0)	0.8 (1.6)	0.02
**Animals ever roam free in household:**											
Chicken/duck	30	19	4.3 (1.2)	3.1 (1.6)	0.01	2.8 (1.3)	2.0 (1.1)	0.03	12.8 (26.2)	6.2 (14.8)	0.42
Cattle/buffalo	13	36	4.3 (1.2)	3.7 (1.6)	0.25	2.8 (1.3)	2.4 (1.3)	0.31	20.5 (36.7)	6.6 (13.3)	0.38
Goat/sheep	17	32	4.4 (1.3)	3.5 (1.5)	0.04	3.1 (1.4)	2.2 (1.1)	0.04	18.1 (33.2)	6.0 (12.6)	0.26
Any animal	31	18	4.3 (1.2)	3.1 (1.6)	0.01	2.8 (1.3)	2.0 (1.1)	0.05	12.6 (25.8)	6.2 (15.3)	0.27
**Animals ever roam free in compound:**											
Chicken/duck	32	17	4.2 (1.2)	3.1 (1.7)	0.02	2.7 (1.3)	2.1 (1.1)	0.06	12.3 (25.4)	6.4 (15.9)	0.20
Cattle/buffalo	14	35	4.1 (1.3)	3.7 (1.6)	0.56	2.7 (1.4)	2.4 (1.2)	0.58	17.3 (35.8)	7.5 (13.9)	0.93
Goat/sheep	21	28	4.2 (1.4)	3.5 (1.5)	0.11	2.9 (1.4)	2.2 (1.1)	0.10	15.3 (30.4)	6.5 (13.3)	0.61
Any animal	36	13	4.2 (1.3)	2.9 (1.6)	0.01	2.7 (1.3)	1.9 (0.9)	0.04	11.3 (4.0)	7.6 (18.6)	0.35
**Animals kept inside home at night:**											
Chicken/duck	2	27	3.2 (1.1)	4.4 (1.3)	0.20	2.5 (0.6)	2.8 (1.4)	0.80	50.7 (69.8)	10.6 (21.2)	0.28
Cattle/buffalo	3	21	5.6 (0.5)	4.0 (1.4)	0.05	4.2 (1.2)	2.7 (1.2)	0.09	11.0 (15.1)	17.8 (31.9)	0.40
Goat/sheep	8	13	5.1 (0.8)	3.6 (2.4)	0.02	3.5 (1.3)	2.5 (1.4)	0.19	5.9 (9.9)	20.4 (37.4)	0.72
Any animal	9	26	4.8 (1.2)	3.9 (1.4)	0.08	3.3 (1.2)	2.6 (1.3)	0.15	16.4 (32.7)	12.4 (23.4)	0.48
**Animal feces observed on floor:**											
Chicken/duck	2	47	5.9 (0.0)	3.7 (1.5)	0.03	5.2 (0.1)	2.4 (1.2)	0.02	23.7 (6.6)	9.9 (23.2)	0.06
Cattle/buffalo	2	47	4.5 (0.7)	3.8 (1.5)	0.65	2.5 (0.6)	2.5 (1.3)	0.88	1.1 (0.2)	10.9 (23.3)	0.96
Goat/sheep	1	48	5.9 (--)	3.8 (1.5)	0.13	5.3 (--)	2.4 (1.2)	0.09	28.4 (--)	10.1 (23.0)	0.16
Any animal	4	45	5.4 (0.9)	3.7 (1.5)	0.02	4.0 (1.3)	2.4 (1.2)	0.03	6.6 (8.3)	10.9 (23.9)	0.25

SD: Standard deviation; MW: Mann-Whitney U-test

Table S5. Number of animals owned by quartiles of generic and cefotaxime-resistant *E. coli* counts and percent abundance of cefotaxime-resistant *E. coli* on floor swabs

**Abundance quartile**		**Chicken/duck**	**Cattle/buffalo**	**Goat/sheep**	**Any animal**
**Owned by household**	**N**	**Mean (SD)**	**Mean (SD)**	**Mean (SD)**	**Mean (SD)**
Generic *E. coli*					
Non-detect	3	0.0 (0.0)	0.0 (0.0)	0.0 (0.0)	0.0 (0.0)
Bottom tertile (1.5-3.3)	16	9.1 (16.1)	1.5 (2.7)	2.1 (4.5)	12.7 (20.6)
Middle tertile (3.5-4.8)	15	10.4 (13.6)	1.1 (1.9)	1.1 (2.0)	12.6 (15.6)
Top tertile (4.9-5.9)	15	21.8 (37.1)	2.8 (2.5)	2.3 (2.2)	26.9 (39.1)
Trend test p-value		0.01	0.02	0.05	0.004
Cefotaxime-resistant *E. coli*					
Non-detect	15	5.5 (8.5)	1.2 (2.3)	1.1 (2.1)	7.9 (11.8)
Bottom tertile (1.5-2.7)	12	9.5 (17.5)	0.6 (1.2)	2.2 (4.9)	12.3 (21.9)
Middle tertile (2.7-3.4)	11	14.5 (12.9)	2.7 (3.1)	1.3 (2.1)	18.5 (13.9)
Top tertile (3.5-5.3)	11	24.7 (43.8)	2.4 (2.4)	2.5 (2.5)	29.6 (49.6)
Trend test p-value		0.02	0.04	0.10	0.01
Percent abundance					
Zero	12	6.9 (9.0)	1.6 (2.5)	1.4 (2.3)	9.9 (12.5)
Bottom tertile (0.1-1.3%)	12	9.5 (9.6)	2.0 (2.6)	1.3 (1.4)	12.8 (11.7)
Middle tertile (1.5-6.3%)	11	17.7 (20.1)	1.4 (2.2)	2.8 (5.3)	21.9 (24.6)
Top tertile (8.9-100%)	11	21.5 (44.3)	2.2 (2.7)	2.0 (2.5)	25.6 (50.0)
Trend test p-value		0.33	0.48	0.52	0.22
**Owned by compound**		**Mean (SD)**	**Mean (SD)**	**Mean (SD)**	**Mean (SD)**
Generic *E. coli*					
Non-detect	3	0.0 (0.0)	0.0 (0.0)	2.7 (4.6)	2.7 (4.6)
Bottom tertile (1.5-3.3)	16	9.8 (16.1)	2.0 (3.7)	2.1 (4.5)	14.0 (21.2)
Middle tertile (3.5-4.8)	15	12.9 (13.4)	2.2 (2.4)	1.3 (2.1)	16.4 (14.8)
Top tertile (4.9-5.9)	15	22.7 (37.2)	2.9 (2.6)	2.1 (2.0)	27.7 (39.1)
Trend test p-value		0.01	0.02	0.25	0.01
Cefotaxime-resistant *E. coli*					
Non-detect	15	5.6 (9.2)	1.9 (3.6)	1.7 (2.7)	9.1 (13.7)
Bottom tertile (1.5-2.7)	12	12.5 (17.2)	1.5 (1.8)	2.2 (4.9)	16.2 (21.1)
Middle tertile (2.7-3.4)	11	15.7 (14.1)	2.7 (3.1)	1.3 (2.1)	19.7 (15.5)
Top tertile (3.5-5.3)	11	25.8 (43.3)	3.0 (2.8)	2.5 (2.1)	31.4 (45.3)
Trend test p-value		0.02	0.08	0.14	0.01
Percent abundance					
Zero	12	7.0 (9.8)	2.3 (3.9)	1.4 (2.3)	10.8 (14.9)
Bottom tertile (0.1-1.3%)	12	12.8 (11.8)	2.3 (2.5)	1.3 (1.4)	16.3 (13.7)
Middle tertile (1.5-6.3%)	11	18.7 (19.3)	2.1 (2.4)	2.8 (5.3)	23.6 (23.2)
Top tertile (8.9-100%)	11	22.5 (43.9)	2.8 (3.0)	2.0 (2.1)	27.4 (47.9)
Trend test p-value		0.21	0.43	0.34	0.11

Table S6. Prevalence and log10-transformed most probable number (MPN) of *E. coli* on child hands by animal ownership and management practices

	N		Prevalence % (n)		Chi2	Log10 MPN Mean (SD)		MW
	**Yes**	**No**	**Yes**	**No**	**p-value**	**Yes**	**No**	**p-value**
Soil floors	19	17	57.9 (11)	47.1 (8)	0.52	1.6 (1.1)	1.0 (0.9)	0.14
**Household owns at least 1:**								
Chicken/duck	20	16	60.0 (12)	43.8 (7)	0.33	1.5 (1.0)	1.1 (1.1)	0.24
Cattle/buffalo	16	20	68.8 (11)	40.0 (8)	0.09	1.6 (1.0)	1.1 (1.0)	0.08
Goat/sheep	12	24	66.7 (8)	45.8 (11)	0.24	1.5 (1.0)	1.2 (1.0)	0.26
Any animal	24	12	58.3 (14)	41.7 (5)	0.35	1.3 (1.0)	1.2 (1.2)	0.51
**Compound owns at least 1:**								
Chicken/duck	22	14	59.1 (13)	42.9 (6)	0.34	1.4 (1.0)	1.1 (1.1)	0.27
Cattle/buffalo	20	16	65.0 (13)	37.5 (6)	0.10	1.5 (1.0)	1.0 (1.1)	0.10
Goat/sheep	14	22	57.1 (8)	50.0 (11)	0.68	1.4 (1.0)	1.3 (1.1)	0.67
Any animal	27	9	55.6 (15)	44.4 (4)	0.56	1.3 (0.9)	1.3 (1.3)	0.82
**Animals ever roam free in household:**								
Chicken/duck	21	15	57.1 (12)	46.7 (7)	0.54	1.4 (1.0)	1.1 (1.1)	0.29
Cattle/buffalo	7	29	71.4 (5)	48.3 (14)	0.27	1.7 (0.9)	1.2 (1.0)	0.17
Goat/sheep	10	26	70.0 (7)	46.2 (12)	0.20	1.6 (1.0)	1.2 (1.0)	0.16
Any animal	22	14	59.1 (13)	42.9 (6)	0.34	1.4 (1.0)	1.1 (1.1)	0.27
**Animals ever roam free in compound:**								
Chicken/duck	23	13	56.5 (13)	46.2 (6)	0.55	1.4 (1.0)	1.2 (1.1)	0.43
Cattle/buffalo	8	28	75.0 (6)	46.4 (13)	0.15	1.5 (0.9)	1.2 (1.1)	0.30
Goat/sheep	12	24	58.3 (7)	50.0 (12)	0.64	1.4 (1.1)	1.2 (1.0)	0.50
Any animal	25	11	56.0 (14)	45.5 (5)	0.56	1.3 (1.0)	1.3 (1.2)	0.65
**Animals kept inside home at night [a]:**								
Chicken/duck	0	20	--	60.0 (12)	--	--	1.5 (1.0)	--
Cattle/buffalo	1	15	100.0 (1)	73.3 (11)	--	0.4 (--)	1.7 (1.0)	--
Goat/sheep	4	8	25.0 (1)	87.5 (7)	--	0.9 (1.0)	1.8 (0.3)	--
Any animal	4	20	25.0 (1)	65.0 (13)	--	0.9 (1.0)	1.4 (0.9)	--
**Animal feces observed on floor [a]:**								
Chicken/duck	1	35	0.0 (0)	54.3 (19)	--	0.4 (--)	1.3 (1.0)	--
Cattle/buffalo	0	36	--	52.8 (19)	--	--	1.3 (1.0)	--
Goat/sheep	0	36	--	52.8 (19)	--	--	1.3 (1.0)	--
Any animal	3	33	33.3 (1)	54.6 (18)	--	1.1 (1.2)	1.3 (1.0)	--

SD: Standard deviation; MW: Mann-Whitney U-test.

[a] No statistical test performed because of small cells.

Table S7. Number of animals owned by quartiles of *E. coli* counts on child hands

**Abundance quartile**		**Chicken/duck**	**Cattle/buffalo**	**Goat/sheep**	**Any animal**
**Owned by household**	**N**	**Mean (SD)**	**Mean (SD)**	**Mean (SD)**	**Mean (SD)**
Generic *E. coli*					
Non-detect	17	5.8 (8.7)	0.8 (1.5)	0.8 (1.8)	7.4 (11.4)
Bottom tertile (1.0-1.6)	7	18.0 (24.6)	1.1 (1.9)	3.3 (6.3)	22.4 (29.8)
Middle tertile (1.9-2.3)	6	14.5 (11.8)	1.5 (1.9)	1.7 (2.9)	17.7 (14.6)
Top tertile (2.4-3.8)	6	40.0 (56.2)	3.8 (3.5)	2.0 (2.4)	45.5 (59.3)
Trend test p-value		0.03	0.02	0.22	0.06
**Owned by compound**	**N**	**Mean (SD)**	**Mean (SD)**	**Mean (SD)**	**Mean (SD)**
Generic *E. coli*					
Non-detect	17	5.6 (8.3)	0.9 (1.5)	1.5 (2.5)	8.1 (10.9)
Bottom tertile (1.0-1.6)	7	18.0 (24.6)	1.1 (1.9)	3.3 (6.3)	22.4 (29.8)
Middle tertile (1.9-2.3)	6	20.5 (6.4)	3.2 (1.9)	1.7 (2.9)	25.3 (6.8)
Top tertile (2.4-3.8)	6	42.0 (55.5)	4.2 (3.6)	1.5 (1.6)	47.7 (58.4)
Trend test p-value		0.01	0.01	0.65	0.02

Table S8. *E. coli* prevalence and abundance on floor swabs by frequency of animal roaming

**Animals roaming frequency**		**Generic *E. coli***		**Cefotaxime-resistant *E. coli***		
**In household**	**N**	**Prevalence % (n)**	**Log10 MPN Mean (SD)**	**Prevalence % (n)**	**Log10 MPN Mean (SD)**	**% abundance Mean (SD)**
Chicken/duck						
Never	19	84.2 (16)	3.1 (1.6)	52.6 (10)	2.0 (1.1)	6.2 (14.8)
Sometimes	15	100.0 (15)	4.0 (1.2)	86.7 (13)	2.6 (1.1)	14.9 (27.0)
Always	15	100.0 (15)	4.6 (1.2)	73.3 (11)	3.0 (1.5)	10.7 (26.0)
Trend test p-value		0.05	0.003	0.16	0.04	0.78
Cattle/buffalo						
Never	36	91.7 (33)	3.7 (1.6)	63.9 (23)	2.4 (1.3)	6.6 (13.3)
Sometimes	12	100.0 (12)	4.2 (1.2)	83.3 (10)	2.7 (1.3)	21.8 (38.0)
Always	1	100.0 (1)	5.9 (--)	100.0 (1)	4.4 (--)	3.5 (--)
Trend test p-value		0.31	0.14	0.16	0.20	0.33
Goat/sheep						
Never	32	90.6 (29)	3.5 (1.5)	62.5 (20)	2.2 (1.1)	6.0 (12.6)
Sometimes	15	100.0 (15)	4.5 (1.2)	86.7 (13)	3.1 (1.3)	20.3 (34.8)
Always	2	100.0 (2)	4.3 (2.2)	50.0 (1)	2.8 (2.3)	1.8 (2.5)
Trend test p-value		0.23	0.05	0.32	0.07	0.43
Any animal						
Never	18	83.3 (15)	3.1 (1.6)	50.0 (9)	2.0 (1.1)	6.2 (15.3)
Sometimes	30	100.0 (30)	4.2 (1.2)	80.0 (24)	2.7 (1.3)	12.9 (26.1)
Always	1	100.0 (1)	5.9 (--)	100.0 (1)	4.4 (--)	3.5 (--)
Trend test p-value		0.03	0.007	0.02	0.03	0.23
**In compound**	**N**	**Prevalence % (n)**	**Log10 MPN Mean (SD)**	**Prevalence % (n)**	**Log10 MPN Mean (SD)**	**% abundance Mean (SD)**
Chicken/duck				p=0.05		
Never	17	82.4 (14)	3.1 (1.7)	47.1 (8)	2.1 (1.1)	6.4 (15.9)
Sometimes	12	100.0 (12)	4.0 (1.2)	83.3 (10)	2.7 (1.3)	16.8 (30.0)
Always	20	100.0 (12)	4.4 (1.3)	80.0 (16)	2.7 (1.3)	9.6 (22.6)
Trend test p-value		0.03	0.01	0.04	0.10	0.32
Cattle/buffalo				p=0.10		
Never	35	91.4 (32)	3.7 (1.6)	71.4 (25)	2.4 (1.2)	7.5 (13.9)
Sometimes	9	100.0 (9)	3.6 (1.0)	44.4 (4)	2.2 (1.3)	12.0 (33.0)
Always	5	100.0 (5)	4.9 (1.4)	100.0 (5)	3.8 (1.1)	26.7 (42.6)
Trend test p-value		0.30	0.22	0.71	0.17	0.51
Goat/sheep				p=0.70		
Never	28	89.3 (25)	3.5 (1.5)	64.3 (18)	2.2 (1.1)	6.5 (13.3)
Sometimes	11	100.0 (11)	4.0 (1.5)	72.7 (8)	2.8 (1.6)	16.2 (31.6)
Always	10	100.0 (10)	4.4 (1.3)	80.0 (8)	3.0 (1.3)	14.2 (30.6)
Trend test p-value		0.16	0.08	0.34	0.09	0.42
Any animal				p=0.07		
Never	13	76.9 (10)	2.9 (1.6)	46.2 (6)	1.9 (0.9)	7.6 (18.6)
Sometimes	32	100.0 (32)	4.1 (1.3)	75.0 (24)	2.6 (1.4)	9.4 (19.8)
Always	4	100.0 (4)	4.6 (1.5)	100.0 (4)	3.4 (0.7)	26.3 (49.2)
Trend test p-value		0.01	0.01	0.02	0.02	0.21

Table S9. *E. coli* prevalence and abundance on child hands by frequency of animal roaming

**Animals roaming frequency**	**In household**			**In compound**		
	**N**	**Prevalence % (n)**	**Log10 MPN Mean (SD)**	**N**	**Prevalence % (n)**	**Log10 MPN Mean (SD)**
Chicken/duck						
Never	15	47.7 (7)	1.1 (1.1)	13	46.2 (6)	1.2 (1.1)
Sometimes	9	55.6 (5)	1.5 (1.1)	7	57.1 (4)	1.6 (1.2)
Always	12	58.3 (7)	1.4 (0.9)	16	56.3 (9)	1.3 (0.9)
Trend test p-value		0.55	0.34		0.60	0.59
Cattle/buffalo						
Never	29	48.3 (14)	1.2 (1.0)	28	46.4 (13)	1.2 (1.1)
Sometimes	6	83.3 (5)	1.9 (0.8)	6	83.3 (5)	1.6 (0.8)
Always	1	0.0 (0)	0.4 (--)	2	50.0 (1)	1.4 (1.4)
Trend test p-value		0.59	0.42		0.31	0.40
Goat/sheep						
Never	26	46.2 (12)	1.2 (1.0)	24	50.0 (12)	1.2 (1.0)
Sometimes	8	75.0 (6)	1.8 (1.1)	5	100.0 (5)	1.4 (1.4)
Always	2	50.0 (1)	0.9 (0.6)	7	100.0 (7)	1.5 (0.9)
Trend test p-value		0.34	0.39		0.42	0.43
Any animal						
Never	14	42.9 (6)	1.1 (1.1)	11	45.5 (5)	1.3 (1.2)
Sometimes	21	61.9 (13)	1.5 (1.0)	23	56.5 (13)	1.3 (1.0)
Always	1	0.0 (0)	0.4 (--)	2	50.0 (1)	1.4 (1.4)
Trend test p-value		0.60	0.48		0.65	0.66

Table S10. *E. coli* prevalence and abundance on floor swabs by animal cohabitation intensity

		**Generic *E. coli***		**Cefotaxime-resistant *E. coli***		
**Animal cohabitation**	**N**	**Prevalence % (n)**	**Log10 MPN Mean (SD)**	**Prevalence % (n)**	**Log10 MPN Mean (SD)**	**% abundance Mean (SD)**
Chicken/duck						
None owned by compound	18	83.3 (15)	3.1 (1.6)	44.4 (8)	2.0 (1.1)	6.0 (15.4)
None owned by household	20	85.0 (17)	3.2 (1.6)	55.0 (11)	2.0 (1.1)	5.6 (14.4)
Kept outside home at night	27	100.0 (27)	4.4 (1.3)	77.8 (21)	2.8 (1.4)	10.6 (21.2)
Kept inside home at night	2	100.0 (2)	3.2 (1.1)	100.0 (2)	2.5 (0.6)	50.7 (69.8)
Trend test p-value		0.02	0.03	0.02	0.05	0.15
Cattle/buffalo						
None owned by compound	20	85.0 (17)	3.4 (1.7)	55.0 (11)	2.3 (1.3)	4.2 (6.0)
None owned by household	25	88.0 (22)	3.5 (1.5)	60.0 (15)	2.2 (1.2)	3.5 (5.5)
Kept outside home at night	21	100.0 (21)	4.0 (1.4)	76.2 (16)	2.7 (1.2)	17.8 (7.0)
Kept inside home at night	3	100.0 (3)	5.6 (0.5)	100.0 (3)	4.2 (1.2)	8.7 (15.1)
Trend test p-value		0.05	0.03	0.07	0.04	0.28
Goat/sheep						
None owned by compound	26	92.3 (24)	3.6 (1.5)	65.4 (17)	2.2 (1.1)	6.4 (2.8)
None owned by household	28	89.3 (25)	3.6 (1.5)	64.3 (18)	2.2 (1.1)	6.8 (13.5)
Kept outside home at night	13	100.0 (13)	3.6 (1.4)	61.5 (8)	2.5 (1.4)	20.4 37.4)
Kept inside home at night	8	100.0 (8)	5.1 (0.8)	100.0 (8)	3.4 (1.3)	5.9 (9.9)
Trend test p-value		0.28	0.06	0.21	0.07	0.49
Any animal						
None owned by compound	11	81.8 (9)	3.1 (1.6)	36.4 (4)	1.8 (0.9)	0.8 (1.6)
None owned by household	14	78.6 (11)	3.1 (1.6)	50.0 (7)	1.8 (0.8)	1.1 (0.5)
Kept outside home at night	26	100.0 (26)	3.9 (1.4)	69.2 (18)	2.6 (1.3)	12.4 (4.6)
Kept inside home at night	9	100.0 (9)	4.8 (1.2)	100.0 (9)	3.3 (1.2)	16.4 (32.7)
Trend test p-value		0.02	0.01	0.01	0.01	0.04

Table S11. *E. coli* prevalence and abundance on child hands by animal cohabitation intensity

		Generic *E. coli*	
**Animal cohabitation**	**N**	**Prev (n)**	**Mean (SD)**
Chicken/duck			
None owned by compound	14	42.9 (6)	1.1 (1.1)
None owned by household	16	43.8 (7)	1.1 (1.1)
Kept outside home at night	20	60.0 (12)	1.5 (1.0)
Kept inside home at night	0	--	--
Trend test p-value		0.42	0.31
Cattle/buffalo			
None owned by compound	16	37.5 (6)	1.0 (1.1)
None owned by household	20	40.0 (8)	1.1 (1.0)
Kept outside home at night	15	73.3 (11)	1.7 (1.0)
Kept inside home at night	1	0.0 (0)	0.4 (--)
Trend test p-value		0.14	0.13
Goat/sheep			
None owned by compound	22	50.0 (11)	1.3 (1.1)
None owned by household	24	45.8 (11)	1.2 (1.0)
Kept outside home at night	8	87.5 (7)	1.8 (0.9)
Kept inside home at night	4	25.0 (1)	0.9 (1.0)
Trend test p-value		0.73	0.69
Any animal			
None owned by compound	9	44.4 (4)	1.3 (1.3)
None owned by household	12	41.7 (5)	1.3 (1.0)
Kept outside home at night	20	65.0 (13)	1.4 (0.9)
Kept inside home at night	4	25.0 (1)	0.9 (1.0)
Trend test p-value		0.88	0.97

Table S12. *E. coli* prevalence and abundance on floor swabs and child hands by cross-categories of household floor type and animal ownership

	**Soil floor**		**Concrete floor**		
	**Animals**	**No animals**	**Animals**	**No animals**	**p-value [a]**
**Floor swabs**	**N = 22**	**N = 6**	**N = 13**	**N = 8**	
Generic *E. coli*					
Prevalence, % (n)	100.0 (22)	100.0 (6)	100.0 (13)	62.5 (5)	0.001
Log10 MPN, mean (SD)	4.8 (1.0)	4.5 (1.0)	3.1 (1.2)	2.0 (0.8)	0.001
Cefotaxime-resistant *E. coli*					
Prevalence, % (n)	90.9 (20)	83.3 (5)	53.9 (7)	25.0 (2)	0.003
Log10 MPN, mean (SD)	3.3 (1.2)	2.6 (0.8)	1.9 (1.1)	1.3 (0.1)	0.001
Percent abundance [b], mean (SD)	14.5 (28.8)	1.3 (1.8)	11.6 (20.0)	0.9 (1.4)	0.19
**Child hands**	**N = 13**	**N = 6**	**N = 11**	**N = 6**	
Generic *E. coli*					
Prevalence, % (n)	53.8 (19)	66.7 (4)	63.6 (7)	16.7 (1)	0.25
Log10 MPN, mean (SD)	1.4 (1.0)	1.9 (1.4)	1.3 (0.9)	0.6 (0.4)	0.24

MPN: Most probable number, SD: Standard deviation.

[a] p-value from chi2 test for prevalence and Kruskal-Wallis test for log10 MPN and percent abundance.

[b] Ratio of MPN counts from IDEXX trays with vs. without cefotaxime supplementation for a given sample.
